# The Relative Risk of Homelessness among Persons with a Disability: New Methods and Policy Insights

**DOI:** 10.3390/ijerph16224304

**Published:** 2019-11-06

**Authors:** Andrew Beer, Emma Baker, Laurence Lester, Lyrian Daniel

**Affiliations:** 1 University of South Australia Business School, Adelaide 5000, Australia; 2 School of Architecture and Built Environment, The University of Adelaide, Adelaide 5005, Australia; emma.baker@adelaide.edu.au (E.B.); laurence.lester@adelaide.edu.au (L.L.); lyrian.daniel@adelaide.edu.au (L.D.)

**Keywords:** disability, housing, homelessness, risk

## Abstract

This paper reports on the first phase of an ambitious program of research that seeks to both understand the risk of homelessness amongst persons with a disability in Australia and shed light on the impact of a significant policy reform—the introduction of the National Disability Insurance Scheme (NDIS)—in changing the level of homelessness risk. This first paper, reports on the level of homelessness risk for persons with a disability prior to the introduction of the NDIS, with a subsequent paper providing updated data and analysis for the period post the implementation of the NDIS. In one sense, this paper provides the ‘base’ condition prior to the introduction of the NDIS but also serves a far broader role in advancing our understanding of how disability and chronic ill-health affects the risk of homelessness. Our research finds that in the period prior to the introduction of the NDIS, a large proportion of people with disabilities were at risk of homelessness, but those whose disabilities affected their schooling or employment were at the greatest risk.

## 1. Introduction

Disability is widespread across the Australian population, with almost 20 per cent of people (4.3 million Australians) having some form of disability [1]. The sheer size of the population affected by disability has substantial implications for housing—its supply, characteristics, and adaptability. People with disabilities are also, importantly, over-represented among the homeless in Australia. Persons with a disability (and also their family members with care responsibilities) are at risk of homelessness because of low incomes, limited engagement with the labour market and restricted capacity within the private rental sector [2]. Importantly though, persons with a disability are not a uniform group and their pathways into homelessness—potential and experienced—will vary by disability type, location and the severity of their disability. It is therefore essential to develop a much stronger understanding of the intersection between disability and homelessness.

This paper reports on the first phase of a program of research that seeks to understand the risk of homelessness amongst persons with a disability in Australia and shed light on the impact of a significant current policy reform—the introduction of the National Disability Insurance Scheme (NDIS)—in changing the level of this risk. Currently being rolled out nationally, the NDIS will be the largest disability support intervention ever undertaken in Australia, with an annual estimated budget in its first year of $22 billion [3]. It is structured to provide individual-focused housing, medical and care support to people with permanent and significant disability. This paper reports on the relative risk of homelessness for persons with a disability prior to the introduction of the NDIS. A subsequent paper will provide updated data and analysis for the period post the implementation of the NDIS. In one sense, this paper provides the ‘base condition’ prior to the introduction of the NDIS, but also serves a far broader role in advancing our understanding of how disability and chronic ill-health affects the risk of homelessness.

### Homelessness and Disability in Australia

There is now an emerging body of work on housing for people with disability. In Australia, 17 per cent of individuals aged between 15 and 64 are living with disability or a long-term health condition [1]. Research has shown that people with disability have poorer housing outcomes than the Australian population as a whole, with significant differences associated with the type of disability, the way in which the disability was acquired and the severity of disability [2,4,5]. Further work has highlighted the differential outcomes between men and women [6,7], and the significant disadvantage experienced by Aboriginal and Torres Strait Islanders [8]. Households in which one or more members have a disability frequently find it difficult to secure appropriately located accommodation, and often therefore live on the urban fringe, in less expensive regional communities at some distance from public transport and other services [4]. For instance, Tually et al. [9] documented the challenges of social exclusion associated with outer suburban communities with a significant concentration of people with disability.

The provision of housing for people with disability has profoundly changed over the past 20 years with the introduction of a long sequence of policy reforms to the funding and delivery of social services across Australia [10]. In recent years, all aspects of disability service provision have been reshaped by the rise of a ‘rights-based’ approach to support [11]. At the beginning of the 21st century, support for people with disability was provided through a mix of government funded programs [12], with services largely funded by states and territories and some support from the Australian Government via the Commonwealth State and Territory Disability Agreement (CSTDA). Access to services was however rationed, with many individuals and families provided with no, or very little, support [13]. State government supported institutions met the needs of those with the most acute disabilities—often within an institutional setting [10]—and specialist not-for-profit disability organisations provided assistance to both persons with a disability and their carers. Unpaid family and other carers were pivotal in the provision of both assistance and housing for people with disability, with informal care replacement costs estimated at $60.3 billion in 2015 [14]. This set of arrangements generated significant challenges to policy and program delivery—including the fact that the CSTDA provided support for only a small proportion of people with disability—resulting in additional cost burdens for state governments, and depriving many people with significant needs of the support they required [12,15].

Families providing informal care assist in supporting much of this unmet demand; however, reduced household incomes, higher medical and other disability-related expenses present additional challenges in securing appropriate and well-located housing [2,16,17]. The reliance on family carers to provide other housing options, however, generated considerable long-term uncertainty, but state and territory-provided group homes and associated congregate living arrangements were a poor match for the needs and preferences of many [18]. Additionally, many ageing parents of people with disability were concerned about where and how their family members would be accommodated when they were less able to provide care and secure housing.

The reality of Australia’s increasingly unaffordable housing market and limited disability support up to the first decade of the 21st century has meant that individuals with a disability have often been presented with few, if any, options with respect to their housing, and have poor outcomes that contribute to the risk of homelessness. Individuals with a disability who leave the family home are commonly directed to non-private dwellings such as residential care homes and boarding houses. From their earlier study of housing careers undertaken in Australia, Beer and Faulkner [19] reported that persons with a disability were less likely to live in a family household that included children and were more likely to live with another person. Almost 40 per cent of individuals with a disability in this study were housed in the social rented sector. The remaining 60 per cent were spread between home ownership and a wide range of other housing tenure types. In concluding their study, Beer and Faulkner suggested that the housing careers of persons with a disability varied significantly by disability type. Individuals with cognitive impairments often exhibited stable housing careers and many were dependent upon family provision throughout their lives. However, the housing of persons who acquired a brain injury later in life would be very different to those of individuals born with a developmental disability.

Homelessness pathways research has, to date, largely ignored the trajectories into homelessness likely to be followed by persons living with disability. Chamberlain and Mackenzie [20] argued that the pathways into homelessness affecting persons with a disability are different to those of the general population, but failed to fully articulate the pathways. The processes and conditions that place people at risk of homelessness embrace structural, institutional and individual factors. Structural factors include poverty, lack of appropriate paid work or the absence of affordable housing options. Institutional factors include the social frameworks and practices that drive individuals into homelessness, or increase their risk of homelessness. This includes policies and legislation preventing persons under the age of 18 from holding a tenancy, the practice of excluding some persons from priority housing, and the waiting list criteria applied by social landlords. Individual factors also contribute; the unique and personal struggles that may pattern a person’s life may include incidences of abuse, violence, or adverse childhood experiences. Non-psychiatric disability could be seen to constitute a previously unrecognised structural factor in the incidence of homelessness across Australia.

The Australian Census of Population and Housing undertaken at the time of these changes [21] provides an invaluable snapshot of the level and incidence of homelessness in Australia at the start of the 21st century. However, this comprehensive source is of limited value in understanding how the incidence of homelessness varies by disability type as these data are not collected. Similarly, Australian Government data collected as part of the National Affordable Housing Agreement (NAHA) failed to collect information on the above groups, but did record information on individuals as they entered a homelessness support service and whether they were provided with accommodation. From this data source we know that 54 per cent of requests for help from homeless persons with physical disabilities, and 55 per cent of requests from persons with intellectual disabilities, were not met. Rather, the individuals requesting help were referred to other agencies. Similarly, 46 per cent of requests for help with housing from persons with psychiatric disabilities were not met directly. In Australia, evidence about physical, sensory, intellectual or brain injury amongst the homeless population is primarily drawn from a limited body of academic research. This work shows that often persons with less ‘obvious’ disabilities are unlikely to be identified when they seek housing assistance, commonly because they do not declare their disability. Individuals may also have more than one disability but this co-morbidity may not be identified until after a person has used a service for an extended period of time.

In order to advance our understanding of the prevalence and character of risk of homelessness among the population with a disability or long-term illness, this paper develops an index of the relative risk of homelessness. The index is applied to two large-scale, national datasets to map and compare relative risk across disability type and restriction.

## 2. Materials and Methods

In this paper we examine the prevalence of risk of homelessness amongst populations with a disability. Two large Australian datasets were analysed—the Household, Income and Labour Dynamics in Australia (HILDA) Survey and the Australian Bureau of Statistics’ General Social Survey (GSS). We classify risk of homelessness for the Australian population along a ten-point continuum. Those with the highest relative risk are classified at level 10, and those with the lowest risk were classified at level 1. Importantly, our approach builds on previous research which principally focused on the classification of homelessness (for example [22,23]) and the experiences of populations classified as homeless (for example [24]). We examine the populations with a disability who have experienced homelessness, those who may be at risk or on the edges of homelessness, as well as those who may be protected from the risk of homelessness.

Our preliminary analysis revealed that Australian populations with a disability are distinct from populations without disabilities in terms of their risk of homelessness. Therefore, we focus on the relative risk of homelessness among populations with disability in three ways:

1. Level of restriction (e.g., profound, mild, schooling), GSS analysis;
2. Category of disability (e.g., intellectual, physical), GSS analysis; and
3. Specific disability types (e.g., hearing problem, chronic or recurring pain), HILDA analysis.

The following analysis seeks to assess the level of risk of homelessness amongst the population with a disability based upon an analysis of the HILDA and GSS data sets. These two large, robust and valuable datasets collect ongoing health and housing information about the Australian population.

### 2.1. The Data Sets

The GSS is an ongoing cross-sectional survey conducted by the Australian Bureau of Statistics (ABS) every four years [25]. It collects data on a range of personal and household characteristics of people aged 18 years and over resident in private dwellings, throughout non-remote areas of Australia. This survey is designed to provide reliable estimates at the national level and for each state and territory, and to enable analysis of the relationships between a range of social circumstances and outcomes. This data is of particular value to this analysis as it allows us to examine the relative prevalence of disabilities and long-term health conditions with respect to their severity. In this paper, analysis is based on data from the GSS dataset collected in 2006; again, prior to the introduction of the NDIS.

The HILDA survey is an annual, nationally representative household-based longitudinal survey of around 18,000 individuals. It has been conducted each year since 2001 and now contains 17 available waves of data [26]. The HILDA dataset collects a wide range of data by surveying adult members of participating households every year via face-to-face interviews and a self-completion questionnaire. Importantly for this study, the HILDA dataset allows us to monitor housing costs, residential stability and mobility, and income while comparing population characteristics, as well as type of disability over time. The analysis in this present paper uses waves 1–9 (data collected prior to the introduction of the NDIS).

### 2.2. The Index of Relative Homelessness Risk

We constructed an Index of Relative Homelessness Risk (IRHR) that allows comparison of the relative exposure to homelessness of a range of disability types, as well as the population as a whole, across the two datasets. The index builds upon previous work on housing precariousness by Mallett et al. [27]. An early conception of the IRHR by Baker et al. [28] conceived of the index as a measure of the risk of homelessness, designed to reflect affordable and secure housing. In order to measure the risk of homelessness, a composite index was constructed and designed to reflect the affordability and security of housing. Components of the index are presented in Table 1. The IRHR is constructed as a simple aggregate of the components; this simple aggregate results in a range of 2 to 30, which is then rescaled to form an index with a range of 1 to 10 (or [0:100]), where 1 represents low risk and 10 represents high risk. This index is measured across whole populations. Due to data availability, the index is constructed differently for the longitudinal HILDA data and for the cross-sectional GSS data. The construction of the index from the GSS data follows, to the extent possible, that of the index using the HILDA data. The differences in the construction of the index based on GSS data are primarily in how residential moves, cashflow problems and income are measured. Nonetheless, the index derived from the two datasets is comparable.

Disability is measured across the population using the two datasets: the GSS allowed the measurement of the level of disability restriction and also by category of disability. The level of disability restriction data was classified by the ABS across five categories [25], as detailed below:

- Profound—always needs help/supervision with core activities
- Severe—does not always need help with core activities
- Moderate—has difficulty with core activities
- Mild—uses aids to assist with core activities
- Persons are classified as having a schooling/employment restriction if they have no core activity limitation, are aged 18 to 20 years, and have difficulties with education, or are less than 65 years and have difficulties with employment.

Disability category was defined in the GSS dataset as: sensory (sight, hearing and/or speech), physical, intellectual, or psychological. Within the broader population these groups were unevenly represented; for example, 14 per cent of the sample population were classified as having a sensory disability, 26 per cent having a physical disability, 2 per cent having an intellectual disability, and 5 per cent a psychological disability.

The second dataset, HILDA, was used to measure relative homelessness risk for groups across 15 specific disability types: limited use of arms or fingers, hearing problem, difficulty learning or understanding, speech problem, blackouts, fits, loss of consciousness, limited use of feet or legs, difficulty gripping things, nervous or emotional condition that needs treatment, any condition that restricts physical activity or work, disfigurement or deformity, any mental illness that requires help or supervision, shortness of breath or difficulty breathing, chronic or reoccurring pain, long-term effect of head injury, stroke or other brain damage, and other long-term health conditions or ailments.

While the results of the analyses of the two datasets are not directly comparable (i.e., the different measures of disability capture different characteristics of an individual’s ability, health or wellbeing), they do allow us an important insight into the relation between disability and the risk of homelessness from multiple perspectives.

## 3. Results

### 3.1. Level of Disability Restriction: Insights from the General Social Survey

In the GSS, a total respondent pool of 11,436 individuals were represented in the analysis of disability by limitation/restriction. Within this total respondent pool, just under half (42 per cent) were recorded as having at least one disability or long-term health condition, and the remaining 58 per cent had none. Among those with a disability or long term health condition, the largest category were those with a non-specific disability, followed by individuals with ‘moderate core activity restrictions’, ‘schooling or employment restrictions’, ‘severe core activity restrictions’, ‘profound core activity restrictions’, and lastly, just over 5 per cent had ‘mild core activity restrictions’.

**Figure 1:**
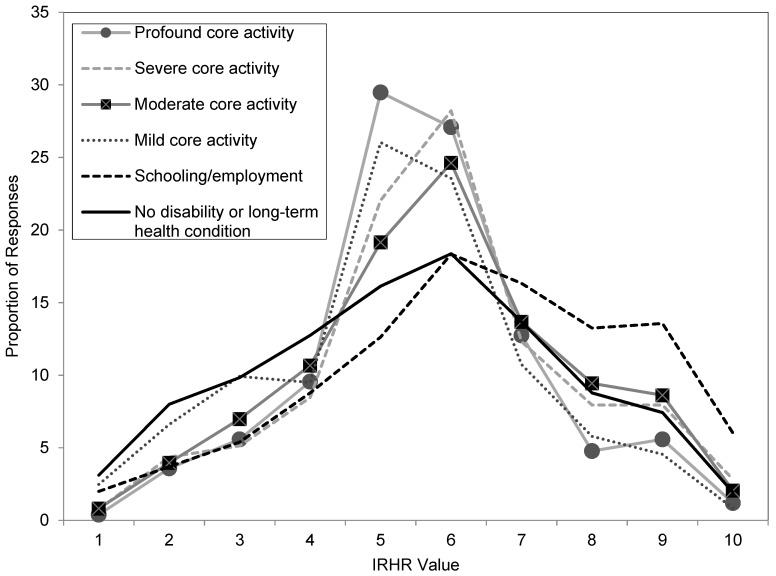
Index of relative homelessness risk score by severity of disability limitation and restriction, by proportion of responses (data source: [25]).

When the characteristics of individuals across this sample were analysed in terms of relative risk of homelessness (as measured by the IRHR), a clear relationship between relative risk of homelessness and the severity of a disability was shown. The strength of this association was statistically confirmed as highly significant (Pr = 0.000). The results are summarised in Figure 1. The figure shows the proportion of persons at each level of disability restriction and by each type of disability by relative homelessness risk score (where 1 is low level risk and 10 is high level risk).

Examining the figure, the IRHR profile of those without disabilities or long-term health conditions (shown as a solid black line) follows a relatively flat ‘normal-type’ curve. Profiles of those with disabilities differ, in the main being much more concentrated in the mid-value IRHR scores. The exception is the group with schooling/employment restrictions (shown as a dashed black line) who are substantially less concentrated in the lower risk IRHRs (1–4), and much more concentrated than the average in the higher risk categories (8–10). Importantly, an individual with a schooling/employment limitation is almost twice as likely (33 per cent) to be in these extreme categories as someone without a limitation (18 per cent).

### 3.2. Disability Category and the Risk of Homelessness: Insights from the General Social Survey

The analysis focuses on four major categories of disability as classified in the GSS—sensory, physical, intellectual, and psychological. In examining psychological disability, we acknowledge that mental illness is not a focus of this research. However, it is a recognised and much researched cause of homelessness and therefore serves as a useful benchmark when examining the interaction between homelessness and other types of disability.

**Figure 2:**
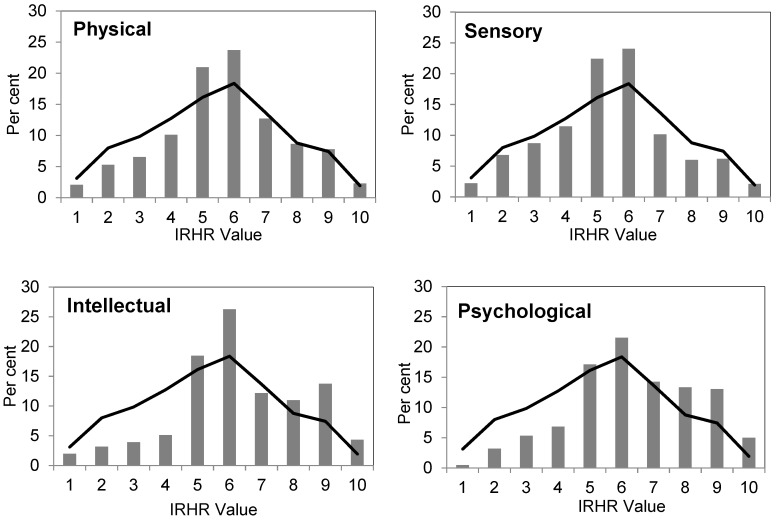
Index of relative homelessness risk for selected disability categories (data source: [25]), grey bars indicate disability and black line indicates no disability or long-term health condition.

For each category of disability we found a highly significant association between relative homelessness risk and disability type (in each case Pr = 0.000). Figure 2 (below) shows the proportions of each population by IRHR score for the four disability types. For comparison, the IRHR category scores for the population with no disability or long-term illness are shown (in black). The analysis highlights the overall difference in relative risk for populations characterised by their intellectual or psychological disabilities, being both under-represented in the low risk categories, and over-represented in the high risk categories. Whereas, the pattern for individuals classified as having physical disabilities is more or less similar to the population with no disability or long-term illness. In comparison, the population with sensory impairments are less commonly represented in the high risk categories than the no disability population, although over-represented in the moderate risk categories.

### 3.3. Disability Type: Insights from the HILDA Survey

**Figure 3:**
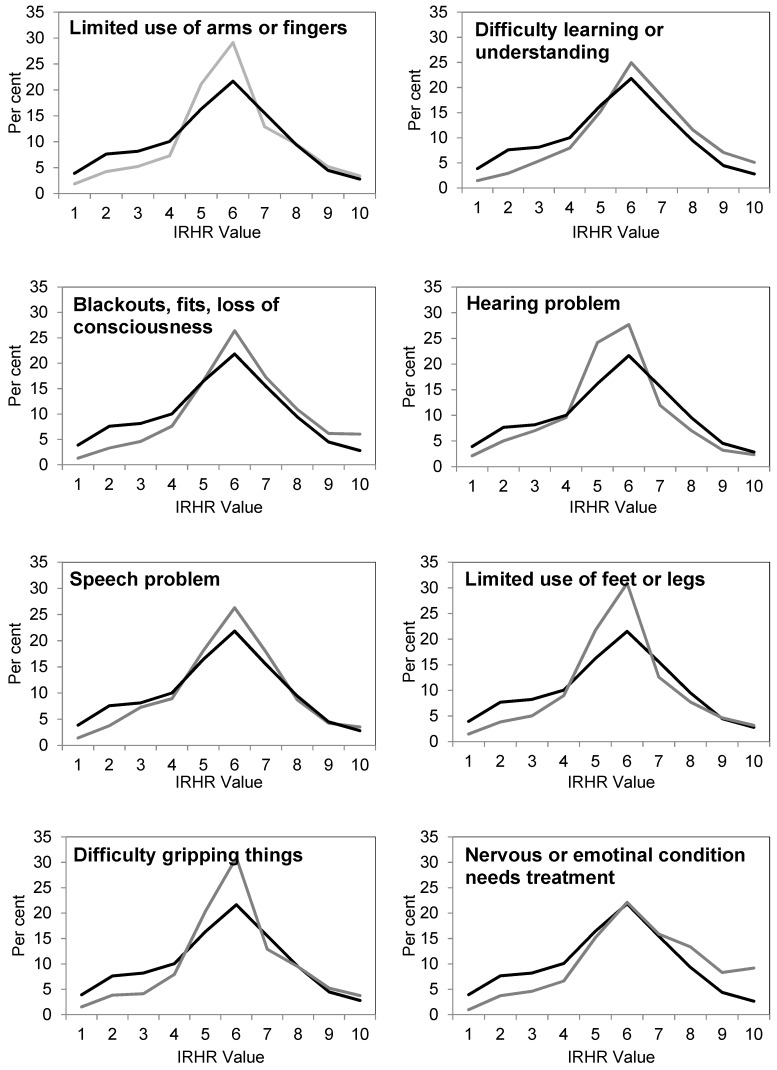

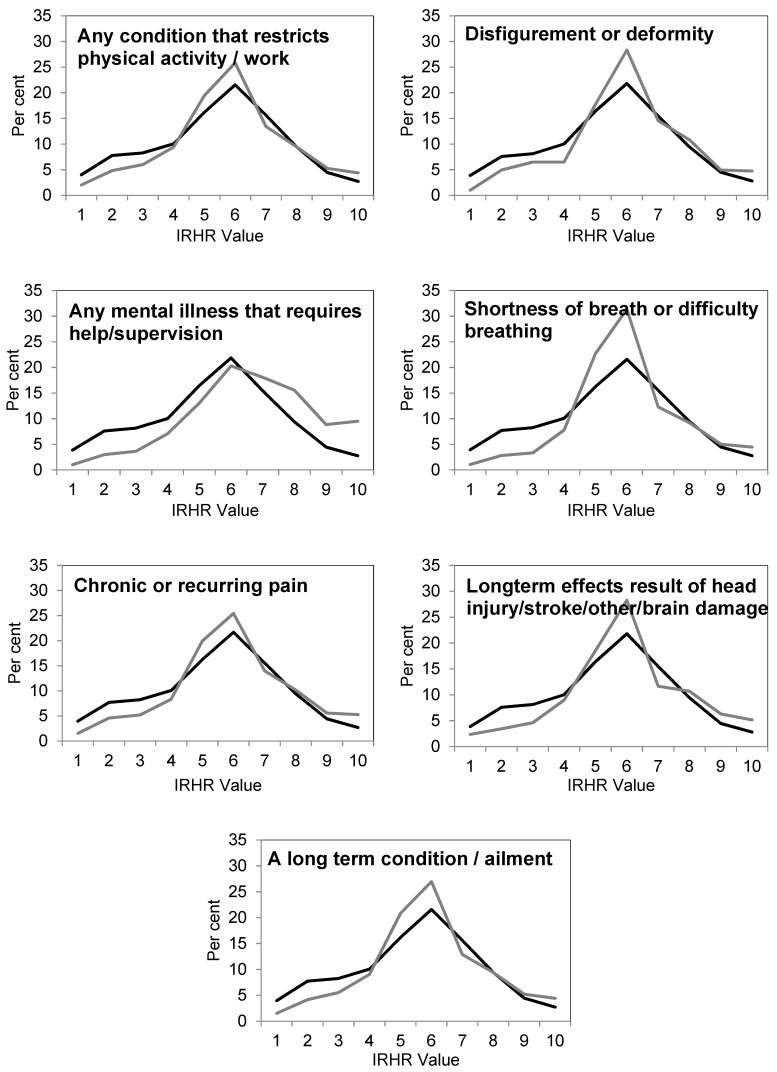
Index of relative homelessness risk by presence (light grey line) and absence (black line) of selected disability types (data source: [26]).

As noted above, HILDA provides important insights into the wellbeing of the broad Australian population and has the potential to shed light on the housing/homelessness circumstances of those with a disability. This analysis of disability type is based upon a pooled analysis of over 120,000 responses to the HILDA survey over the 9 years where the data was available. This large dataset allows us to robustly examine the prevalence of specific disability characteristics by the relative homelessness risk of individuals. The nature of the HILDA data collection allows for greater precision in the specification of disabilities and in the analysis of exposure to risk. The results, comparing relative risk for individuals who had specific disabilities (in grey), with relative risk for individuals without those specific disabilities (in black), are summarised in Figure 3 below.

A number of findings stand out in these graphs: firstly, in the case of each disability type, individuals with a disability are much less likely to have a low relative homelessness risk (IRHR value) than those without a disability. Secondly, with only one exception, individuals with disabilities are over-represented in the mid ranges of relative homelessness risk compared to those without disabilities. Third, those with physical disabilities tend not to be over-represented, compared to individuals without disabilities, in the higher relative risk of homelessness categories. For these types of disability, similar proportions with a disability are in the higher IRHR categories compared to individuals without. For individuals whose disability is related to mental illness or brain injury, there is an increased likelihood of these individuals having higher relative homelessness risk. We highlight here individuals with difficulty learning or understanding, where almost 25 per cent of individuals with this type of disability are in the most extreme levels of homelessness risk (values 8, 9, and 10), compared to only 16 per cent of individuals without that disability. Similarly, for individuals with a mental illness that requires help or supervision, the results are even more extreme, with 34 per cent of this population in extreme homelessness risk, compared to 17 per cent of individuals without this type of disability.

## 4. Discussion

This paper set out to understand the risk of homelessness amongst persons with a disability in Australia in the early- to mid-2000s, prior to the introduction of a generationally important disability policy reform, the NDIS. The paper examined the level of risk for persons with a disability prior to the introduction of the NDIS, and a subsequent paper will provide updated data and analysis for the period post the implementation of the NDIS. In one sense, this paper simply sheds light on the starting condition prior to the introduction of the NDIS but it has also generated its own unique, and valuable insights, into the impact of disability and chronic ill-health on the propensity to experience homelessness.

The analyses presented in this paper have provided confirmation of the underlying assumption of this research: that persons with a disability have a greater exposure to the risk of homelessness than the general population. Importantly however, we showed that the relative risk of homelessness (and hence any resulting policy response) cannot be generalised for the population with a disability because exposure is not evenly distributed amongst those with a disability. Across the analyses, individuals with schooling/employment restrictions, those with psychological and intellectual disabilities, and those with mental illnesses were especially vulnerable to extreme levels of relative homelessness risk. Individuals with each of these restriction types are much more likely than the population without a disability to be at risk of homelessness—of whom approximately one in five were classified at the same extreme level of homelessness risk.

Our analysis suggests that during the study period, disability was strongly associated with the relative risk of homelessness and that, within the disabled population, there were differences in risk depending on the severity of restriction and the type of disability. People with schooling/employment restrictions appear to be highly vulnerable to risk of homelessness. The analysis also revealed much higher levels of relative risk for people with intellectual and psychological disabilities. We speculate that these patterns, at least partially, reflect the differential effect of welfare and policy interventions on the relative homelessness risk of individuals with different forms of disability. Because interventions are focused, and also taken up unevenly across a population, welfare protection is provided, but unevenly. This affects IRHR patterns across and between populations, and we speculate that this contributes to different IRHR profiles between disability types. For example, individuals with physical and sensory disabilities have an IRHR profile that is more similar to the population with no disability, and this leads us to question if this is a reflection of the greater effectiveness of housing interventions for this group. Importantly though, the IRHR profile for each of these disability types would doubtless be very different in a policy environment where no welfare interventions were provided, and mapping the IRHR profile changes due to specific large scale interventions is an area ripe for further research.

It is also important to note the small but likely influence of age. In the populations classified as having either physical of sensory disabilities, a proportion of these persons would have acquired their disability as a result of natural ageing. In these cases, they have experienced a shorter length of exposure to disability, meaning that many would have spent greater periods of their life course in employment, thereby acquiring housing and financial assets. Such shorter exposure length to disability is likely to provide some protection from relative homelessness risk.

Reinforcing the earlier results, this analysis highlights an overall higher relative risk of homelessness for people with mental health and learning related disabilities. Finally, this examination of individual disability types does not capture co-morbidity within the population. Many individuals in our large sample have more than one disability type, and although the effects of co-morbidity have not been included in this study, we acknowledge that this will also influence vulnerability to poorer relative homelessness risk.

Our findings reflected the differentiated risk of homelessness across various disability groups in Australia, which in turn indicate the uneven level of government and NGO-provided support available to persons with different types of disability in Australia in the early 2000s. Overall, the paper highlighted a number of sub-groups within the disabled population who are more vulnerable, and hence, need additional (and especially targeted) support with their housing. Beyond the insights into extreme risk of homelessness, our modelling has emphasised the uneven exposure that some groups have to moderate homelessness risk. These groups (i.e., those with intellectual disabilities, or specific physical disabilities, such as difficulty breathing, and limited use of arms or legs), had a much higher likelihood of classification of a moderate risk of homelessness when compared with those without a disability. This is important because, firstly, these groups tend to be equivalently under-represented in the very low risk categories (representing secure and affordable housing), and also because this reflects housing disadvantage and a potential homelessness precondition, which may not be detected in studies that focus only on homelessness.

## 5. Conclusions

Overall, we can conclude that in Australia in the early 2000s, persons with a disability had greater exposure to the risk of homelessness than the general population. Individuals with some disability types (for example learning difficulties or mental illness) were more vulnerable to homelessness, and hence needed additional (and especially targeted) support with their housing, but often such support was not provided. Finally, we acknowledge that, while this study has highlighted the differential prevalence of risk across the population affected by a disability, it has not shed light on the triggers or buffers that may increase vulnerability or protection from homelessness. The follow-up paper may show that the risk of homelessness for persons with a disability has decreased since the introduction of the NDIS, but we also need to be open to the possibility that the opposite holds.

## Figures and Tables

**Table 1:** Index of relative homelessness risk components by dataset.

Index of Relative Homelessness Risk Components	General Social Survey Variables	Household, Income and Labour Dynamics in Australia Variables
Cash flow	This variable is a count, at each wave, of the number of cash flow problems that are reported (7 in HILDA, 9 in the GSS)	
Number of residential moves	The number of moves in the preceding five years	A cumulative sum of the number of moves undertaken in the previous wave, the count at wave 1 is zero
Evictions	Variable signifying if the individual was evicted from their last accommodation by the landlord	
Low income	Deciles of household income (equivalised gross weekly), reverse coded	Deciles of household income (gross annual income), reverse coded
Housing costs	This variable is constructed using the values for mortgage and rent payments and is structured as deciles of Housing cost	
